# The Expressing Patterns of Opioid Peptides, Anti-opioid Peptides and Their Receptors in the Central Nervous System Are Involved in Electroacupuncture Tolerance in Goats

**DOI:** 10.3389/fnins.2018.00902

**Published:** 2018-12-13

**Authors:** Juan Wan, Zhengying Qiu, Yi Ding, Sha Nan, Mingxing Ding

**Affiliations:** ^1^College of Veterinary Medicine, Huazhong Agricultural University, Wuhan, China; ^2^Lanzhou Institute of Husbandry and Pharmaceutical Sciences of Chinese Academy of Agricultural Sciences (CAAS), Lanzhou, China

**Keywords:** electroacupuncture (EA) tolerance, enkephalin (ENK), cholecystokinin octapeptide (CCK-8), orphanin FQ (OFQ), μ-opioid receptor (MOR), CCKB receptor, opioid receptor-like1 receptor (OPRL1)

## Abstract

To investigate dynamic processes of enkephalin (ENK), cholecystokinin octapeptide (CCK-8), orphanin FQ (OFQ) and their receptors (μ opioid receptor, MOR; CCK B type receptor, CCKBR and opioid receptor-like 1 receptor, OPRL1) in the central nerve system (CNS) during electroacupuncture (EA) tolerance, EA of Sixty Hz was used to stimulate goats for 6 h. Pain threshold was measured using potassium iontophoresis. The expression levels of ENK, CCK-8, and OFQ and their receptors were determined with ELISA and qPCR, respectively. The results showed that the change rates of pain threshold in EA-treated goats decreased from 89.9 ± 11.7% at 0.5 h to –11.4 ± 8.9% at 6 h. EA induced the decreased ENK and increased CCK-8 and OFQ in the most measured nuclei. EA caused decreased preproenkephalin mRNAs in ACB, CAU, PVH, and PAG at 4 h, and decreased or unchanged MOR mRNAs at 2–6 h, but increased CCK mRNAs in CAU, PVT, PVH, PAG, and SCD at 4–12 h. Increased prepronociceptin mRNAs and fluctuated CCKBR and OPLR1 mRNAs were found in the most measured nuclei. ENK levels were positively correlated (*p* < 0.01) with the change rates of pain thresholds in the measured nuclei or areas while CCK-8 levels (or OFQ levels) were negatively correlated (*p* < 0.01) with the pain thresholds in CAU (or CAU and ACB). These results suggest that the development and recovery of EA tolerance may be associated with the specific expression patterns of opioid peptides, anti-opioid peptides and their receptors in the analgesia-related nuclei or areas.

## Introduction

Electroacupuncture (EA), an alternative of traditional acupuncture, has been widely used for treating various pathologic conditions with little side effect, especially pain disorders (such as post-operative pain, inflammatory pain, and neuropathic pain) in animals and humans (Zeng et al., 2016; Hu et al., 2017; Wan et al., 2017). EA in combination with analgesics or anesthetic (acupuncture-assisted anesthesia) reduces the doses of these drugs, shows better analgesic effects and extends its clinic application (Liu et al., 2009; Shah et al., 2016; Cui et al., 2017b). Studies demonstrate that EA stimulation for a short time (10–40 min) can induce analgesic effect (Wang et al., 2008; Zhao, 2008; Hu et al., 2016). However, its prolonged or repeated application will result in a gradual fading away of such hypalgesic effect, which is called “EA tolerance (EAT)” (Han et al., 1979; Han J.S. et al., 1981; Cui et al., 2016, 2017a).

Because the tolerance to EA stimulation results in the decrease or even loss of EA treatment effects, it has attracted more attention from practitioners and researchers. To explore EAT mechanism, some studies focus on the roles of analgesic and anti-analgesic neuromodulators in the central nerve system (CNS). It has been verified that EA exerts its analgesic effect mainly through the release of endogenous opioid peptides (EOPs) (endorphins, enkephalin and dynorphin) in the CNS (He et al., 1985; Chen and Han, 1992b; Han, 2003; Wen et al., 2010). Tang et al. (1979) continually and repeatedly electroacupunctured rats and found that both EA modalities induced the higher levels of EOPs, but at the meantime developed EAT. Studies have shown that EA induces the release of anti-opioid peptides, such as cholecystokinin octapeptide (CCK-8) (Bian et al., 1993) and orphanin FQ (OFQ) (Tian et al., 1998), as it increases levels of opioid peptides in the CNS. The increase in the anti-opioid peptides is believed to be due to the feedback regulation of opioid peptides. The enhanced antagonism of anti-opioid peptides to analgesia may be one of mechanisms underlying EAT (Han, 1997). However, the change patterns of both opioid peptides and antiopioid peptides in the development of EAT have not been clarified yet.

Opioid peptides or anti-opioid peptides exert analgesic or anti-analgesic effect through binding to their corresponding receptors. Therefore, some researchers have paid attention to the roles of these receptors in EAT. Ni et al. (1987) induced EAT using continuous EA and found that the opioid receptors decreased in the brain. Dong et al. (1998) also found the decreased number of opioid receptors in the midbrain and striatum of rats after repeated application of EA. Huang et al. (2007) demonstrated that CCK B receptor (CCKBR) took part in the regulation of EAT through its influence on opioid receptors in mice. Nevertheless, the dynamic changes of opioid and anti-opioid peptide receptors and their relationships with the corresponding ligands in the development of EAT need to be studied.

Studies have shown that EA-induced analgesic effect in goats (ruminants) is superior to that in rats or human (Qin et al., 1996; Han, 1997; Liu et al., 2009). In addition, compared with small experimental animals, goats have larger brains that make some nuclei sampling easier. Thus, ruminants are optimal animal models for studying the mechanisms underlying EA analgesia and EAT. In the present study, goats were continually stimulated with EA for 6 h. The changes of pain thresholds were used to determine the development of EAT. The dynamic expressing processes of enkephalin (ENK, a representative of EOPs), anti-opioid peptides (CCK-8 and OFQ) and their receptors (Mu opioid receptor, CCKBR and opioid receptor-like 1 receptor) in the CNS were determined to explore the mechanism underlying EAT. We hypothesized that the tipping of the balance of opioid and anti-opioid peptides and/or the modifications of their receptors may contribute to the development of EAT.

## Materials and Methods

### Animal Preparation

The study was conducted under the guidelines approved by Institutional Animal Care and Use Committee of the Huazhong Agricultural University, Wuhan, China and adhered to the guidelines of the Committee for Research and Ethical Issues of the International Association for the Study of Pain (Permit number: HZAUGO-2016-005).

Seventy-eight two-year-old hybrid male goats, weighing 30.8 ± 3.1 kg, were purchased from Hubei Agricultural Academy of Science, and fed by a dry grass diet supplemented with a cereal-based concentrate, and drank freely. They were dewormed and accustomed to surroundings for 2 weeks. The experiment was performed in a quiet environment after 12 h of fasting, with a temperature of 20–25°C.

### Electroacupuncture

A set of “Baihui,” “Santai,” “Ergen,” and “Sanyangluo” points (here using the Pinyin Naming System instead of the Meridian Numbering System because animal’s meridians are not completely recorded) were selected, which were traditionally used in veterinary medicine to achieve effective analgesia in cattle (Peng et al., 1982) and goats (Liu et al., 2009). The anatomic location and use of these points have been described in detail in veterinary medicine (Klide and Kung, 1982). The “Baihui” point was identified on the dorsal midline between the spinous processes of the last lumbar and the first sacral vertebrae (but Baihui point in humans is located at the top of the skull). The “Santai” point was identified on the dorsal midline between the spinous processes of the fourth and fifth thoracic vertebrae. The “Ergen” points were identified bilaterally, with each at the pit ventrocaudal to the ear base between the ear base and the cranial border of the transverse process of the atlas on each side. The “Sanyangluo” points were also identified bilaterally, with each at approximately 5 cm ventral to the lateral tuberosity of the radius in the groove between the common digital extensor and the lateral digital extensor muscles of the forelimb. The “Baihui” and “Santai” points on the dorsal midline and the “Ergen” and “Sanyangluo” points on the right side of the body were used in this study. The acupoint sites were shaved and disinfected. The acupuncture needle (0.45 mm in diameter and 5 cm in length) was inserted perpendicularly into the “Baihui” point at a depth of approximately 3 cm. For the “Santai” point, the needle was inserted at a 45° angle at a depth of approximately 4 cm. These two needles shared a pair of wires from one output of the WQ-6F Electronic Acupunctoscope (Beijing Xin dong hua Electronic Instrument Co., Ltd., Beijing, China). One needle (0.45 mm in diameter and 7.5 cm in length) was inserted into the right “Ergen” point to reach the subcutaneous tissue of the right temporal fossa. Another needle was inserted at a 30° angle in a ventromedial direction into the right “Sanyangluo” point to reach the subcutaneous tissue of the medial side of the right forelimb. The two needles shared a pair of wires from another output of the same machine, and the parallel output option was selected. EA parameters were set according to the report by Qiu et al. (2015). The goats were stimulated with EA at 60 Hz; the intensity (3.2 V) remained constant during the entire EA procedure. The goats that were only dealt with needles left in the acupoints without electricity were used as the sham control. The goats in the blank control group were not treated with either needles or electricity. The goats in the blank and sham control groups were restrained as the EA-treated goats for consecutive 6 h, and then unlashed. The needles were withdrawn immediately.

### Determination of the Pain Threshold

The pain threshold was measured on the center of the right flank using potassium iontophoresis (Liu et al., 2009) by a direct current induction therapy apparatus (Shantou Medical Equipment Factory Co., Ltd., Shantou, China). The pain was induced by potassium iontophoresis through a gradual increase in potassium ions (an effective pain stimulus) passing through the skin; the potassium ions into the subcutaneous tissues were positively proportional to the increase in voltage or current. One skilled person who was blinded to the goat assignments assessed the pain threshold. The site of the pain threshold was shaved and cleaned with soap and water, and disinfected with 75% ethanol. Two electrodes were soaked with saturated potassium chloride and placed 1–2 cm apart on the skin. The direct current induction therapy apparatus was used to deliver the pulsed direct current to the electrodes, which forced potassium ions into the subcutaneous tissues. The voltage was increased stepwise. The voltage level was recorded at the moment once the contraction of the local skin and muscle (along with the animal’s head turning toward the abdomen, back hunching and eluding movements) was observed. Then, the current was turned off.

The pain threshold measurements of the goats were taken 3 times immediately before the insertion of the needle or after EA. The mean voltages before and after EA procedures were expressed as V_0_ and V_n_, respectively. The percentage change in the pain threshold was calculated as follows: Δ (%) = (V_n_ – V_0_)/V_0_ × 100%.

### Experimental Procedures

The experiment goats were randomly divided into three treatments: blank control (6 goats), sham control (6 goats), and EA treatment (66 goats).

EAT was established according to the reported by Han J. et al. (1981) and our pretest. To induce EAT, 66 goats in EA group received restraint and continued EA. The needles were withdrawn at 6 h when EA was terminated, and the goats were unlashed. The Blank and Sham goats received the same restraint as EA goats. Pain thresholds were measured at 0, 0.5, 2, 4, and 6 h of the experiment. Six goats randomly taken from 66 goats at 0, 0.5, 2, 4, 6, 12, 18, and 30 h, respectively, were euthanized with intravenous administration of xylidinothiazoline at 3 mg/kg for detecting expression levels of opioid and anti-opioid peptides and their receptors in the brain and spinal cord. To observe EAT recovery, another six goats randomly taken from the left EAT goats at 12, 18, and 30 h, respectively, received re-restraint and EA for 30 min, and their pain thresholds were measured immediately before and after each EA. According to reports of Cheng et al. (2012, 2013), the pain threshold rates, opioid and anti-opioid peptides and their receptors of the Control and Sham goats did not significantly change during their experiments. Therefore, pain thresholds were not observed in the Control and Sham goats after 6 h, and the opioid and anti-opioid peptides and their receptors were only detected in the EA-treated goats to display their dynamic changes in this trial.

### Tissue Collection of the Analgesia-Related Nuclei and Areas

**FIGURE 1:**
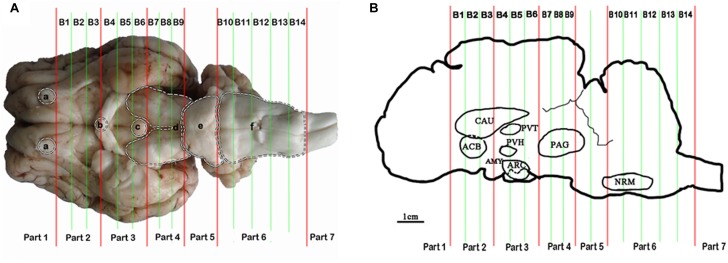
Brain sectioning. **(A)** (a) the residual part of the olfactory bulbs, (b) optic chiasm, (c) mamillary body, (d) cerebral peduncles, (e) pons, and (f) medulla oblongata. **(B)** In the blocks nuclei or areas: the nucleus accumbens (ACB) and the caudate nucleus (CAU) in B2, the paraventricular nucleus of the thalamus (PVT) and the paraventricular nucleus of the hypothalamus (PVH) in B4, the arcuate nucleus (ARC) and the amygdala (AMY) in B5, the periaqueductal gray (PAG) in B8 and the nucleus raphe magnus (NRM) in B13 are located.

Some nuclei and areas, including the nucleus accumbens (ACB), the caudate nucleus (CAU), the paraventricular nucleus of the thalamus (PVT), the paraventricular nucleus of the hypothalamus (PVH), the arcuate nucleus (ARC), the amygdala (AMY), the periaqueductal gray (PAG), the nucleus raphe magnus (NRM), and the spinal cord dorsal horn (SCD), proved to be analgesia-related nuclei and areas (Figure 1B). These nuclei and areas were sampled after 6 h of EA as described by Cheng et al. (2013). Briefly, the goat brains were immediately taken out of the skull. The whole brain tissue was placed laterally in the precooled section mold for goat’s brain. The brain was divided into seven parts using an RNAse-free blade according to the dividing lines (olfactory bulb base edge, optic chiasma center, nipple body posterior margin, the groove between the cerebral peduncle and the pons, the groove between the pons and the medulla oblongata and the boundary between the medulla oblongata and the spinal cord). Then, each among the second to the fourth parts was subdivided into three tissue blocks on average (B1–B9), and the sixth part was subdivided into five tissue blocks (B10–B14) (Figure 1A). Meanwhile the spinal cord from segment L4-6 was obtained. The nuclei and areas from the brain were identified according to the photographic atlas of goat’s brain and the morphological characteristics of the neurons (Tindal et al., 1968, 1987; Dean et al., 1999; Sunagawa et al., 2015). The tissues of bilateral nuclei and areas were collected with 4–10 mm diameters of DEPC-treated plastic tubes (8 and 7 mm tubes for CAU and ACB in B2, 5 mm tubes for PVH and PVT in B4, 5 and 8 mm tubes for ARC and AMY in B5, 10 mm tubes for PAG in B8, and 4 mm tube for NRM in B11, respectively) (Figure 1B). These tissues were frozen immediately, ground into fine powder in liquid nitrogen, and divided into two replicates for subsequent ELISA and qPCR detection, respectively. Before the subsequent experiment, the samples were stored at −80°C.

### Measurement of ENK, CCK-8 and OFQ Levels

The powdered samples were thawed and lysed with 0.01 mM phenylmethanesulfonyl fluoride in PBS (pH 7.4) with 100 mg tissue: 1 mL solution. The mixture was homogenized and centrifuged at 3000 rpm for 15 min. The protein concentration of the supernatant was determined using NanoDrop Spectrophotometer (Thermo Fisher Scientific, Inc., United States). The ENK, CCK-8, and OFQ levels were assayed using Goat enkephalin ELISA Kit (R&D Systems, China), Goat cholecystokinin-8 ELISA Kit (Cusabio Biotech Co., Ltd, China), and Goat OFQ ELISA Kit (Cusabio Biotech Co., Ltd, China), respectively, according to the manufacturer’s instructions.

### Measurement of Gene Expression

The powdered samples were homogenized in buffer RZ solution (RNA simple Total RNA Kit, TIANGEN BIOTECH (BEIJING) CO., China) for total-RNA extraction according to the manufacturer’s instructions. RNA sample (500 ng) was reverse-transcribed using High Capacity RNA-to-cDNA Kit (Applied Biosystems, United States). Quantitative real-time PCR was performed and analyzed according to the instructions of manufacturer (Applied Biosystems Step One Plus^TM^ Real-Time PCR System, ABI Co., United States). PCR products were detected by SYBR Green chemistry. The gene expression levels of preproenkephalin (PENK), CCK, prepronociceptin (PNOC), CCKBR, opioid receptor-like receptor 1 (OPRL1), and Mu opioid receptor (MOR) were measured with 2^−ΔΔCt^ method. The primers were shown in Table 1. According to the pretest, the beta-actin stably expressed in the goat brain compared with GAPDH, therefore, sample cDNAs were normalized with beta-actin. All samples were measured in triplicate.

**Table 1:** The quantitative real-time PCR primers of opioid- and antiopioid- peptide genes.

Genes and sequence reference (Gene Bank accession No.)	Forward primer/Reverse primer	Length of PCR product (bp)
β-actin (AF481159.1)	5′-**TGAACCCCAAAGCCAACC**-3′	159 bp
	5′-**GGCGTACAGGGACAGCA**-3′	
PNOC (XM018051867.1)	5′-**GGTCTTCACCAGCCCTCT**-3′	182 bp
	5′-**CTGTCCTCTTCTGCCTTT**-3′ ′	
OPRL 1 (XM005688252.3)	5′-**AGGCTGTAGCAGATGGAGATGA**-3′	99 bp
	5′-**CCCCACCCCACAGGATTA**-3′	
CCK (XM005695576.3)	5′-**ACATGGGCTGGATGGATTT**-3′	109 bp
	5′-**CCTTTGAGTCAGGAGGTTGC**-3′ ′	
CCKBR (XM018059457.1)	5′-**GCCATCTGCCGACCACT**-3′	245 bp
	5′-**ACCGCCATAACCACCC**-3′	
PENK (XM018058489.1)	5′-**AACTCCTCCAACCTGCTC**-3′	199 bp
	5′-**GACGACCCACTCTTCTCA**-3′	
MOR (XM005684928.3)	5′-**TTCCGCACTCCCCGTAAT**-3′	93 bp
	5′-**TGTCGTTGCCATGAACATCA**-3′	

*CCK, cholecystokinin; PNOC, Prepronociceptin; PENK, Preproenkephalin; CCKBR, Cholecystokinin B receptor; OPRL 1, Opiate receptor-like 1; MOR, μ-opioid receptor).*

**Table 2:** The effect of EA on levels of PENK gene expression in the CNS of goats (2^−ΔΔCt^, mean ± SD, n = 6).

Nuclei and areas	0 h	0.5 h	2 h	4 h	6 h	12 h	18 h	30 h
ACB	1.04 ± 0.10^ab^	0.80 ± 0.30^abc^	0.46 ± 0.24^bc^	0.37 ± 0.29^c^	0.39 ± 0.17^c^	0.45 ± 0.20^bc^	1.12 ± 0.44^a^	1.33 ± 0.53^a^
CAU	1.02 ± 0.12^ab^	1.03 ± 0.15^a^	1.07 ± 0.05^a^	1.09 ± 0.24^a^	0.80 ± 0.08^b^	0.97 ± 0.10^ab^	0.99 ± 0.09^ab^	0.96 ± 0.09^ab^
PVT	1.02 ± 0.14^c^	0.29 ± 0.20^c^	0.37 ± 0.13^c^	0.44 ± 0.16^c^	0.92 ± 0.42^c^	7.67 ± 1.30^a^	0.77 ± 0.29^c^	2.37 ± 0.68^b^
PVH	1.00 ± 0.11^cd^	0.94 ± 0.28^d^	1.45 ± 0.34^bcd^	1.26 ± 0.23^bcd^	1.54 ± 0.32^bcd^	1.47 ± 0.36^bc^	1.65 ± 0.25^b^	2.50 ± 0.32^a^
ARC	1.02 ± 0.10^cd^	1.15 ± 0.13^c^	1.82 ± 0.34^b^	2.86 ± 0.29^a^	0.56 ± 0.10^e^	0.71 ± 0.11^de^	0.75 ± 0.18^de^	1.19 ± 0.18^c^
AMY	0.99 ± 0.10^b^	1.99 ± 0.41^ab^	1.05 ± 0.20^b^	0.69 ± 0.29 ^b^	0.71 ± 0.51^b^	1.17 ± 0.80^b^	2.80 ± 0.59^a^	2.05 ± 1.72^ab^
PAG	1.04 ± 0.07^d^	1.51 ± 0.25^cd^	1.49 ± 0.36^cd^	1.46 ± 0.32^cd^	1.99 ± 0.22^ab^	1.80 ± 0.36^bc^	2.29 ± 0.38^ab^	2.55 ± 0.29^a^
NRM	1.05 ± 0.13^bc^	0.97 ± 0.24^bc^	0.74 ± 0.17^bc^	0.61 ± 0.18^c^	0.87 ± 0.12^bc^	1.15 ± 0.38^ab^	0.96 ± 0.33^bc^	1.66 ± 0.42^a^
SCD	1.01 ± 0.90^b^	1.30 ± 0.68 ^ab^	3.14 ± 1.37^ab^	2.16 ± 0.79^ab^	3.33 ± 1.25^a^	3.60 ± 1.69^a^	3.45 ± 1.44^a^	2.23 ± 1.43^ab^

*The measured nuclei and areas include the nucleus accumbens (ACB), the caudate nucleus (CAU), the paraventricular nucleus of the thalamus (PVT), the paraventricular nucleus of the hypothalamus (PVH), the arcuate nucleus (ARC), the amygdala (AMY), the periaqueductal gray (PAG), the nucleus raphe magnus (NRM) and the spinal cord dorsal horn (SCD). Values with different letters within the same row differ significantly (p < 0.05). One-way ANOVA followed by Bonferroni’s post-test. The symbols have the same meanings in Tables 3–7.*

### Statistical Analysis

Statistical analysis was undertaken with SPSS 17.0 software (SPSS Inc., Chicago, United States). All data are presented as the mean ± SD. All data were tested with Kolmogorov–Smirnov Test for a normal distribution. Pain threshold changes were analyzed with a repeated measure analysis of variance (ANOVA) to detect changes over time and between treatments, and the protein and gene data were subjected to ONE-WAY ANOVA. The Bonferroni test was applied when significant differences were found. The correlation coefficient (Pearson’s) was used to examine the correlations between pain threshold changes and levels of ENK, CCK-8, or OFQ. Statistical significance was evaluated by determining whether *p* < 0.05.

## Results

### The Time-Course of EAT

**FIGURE 2:**
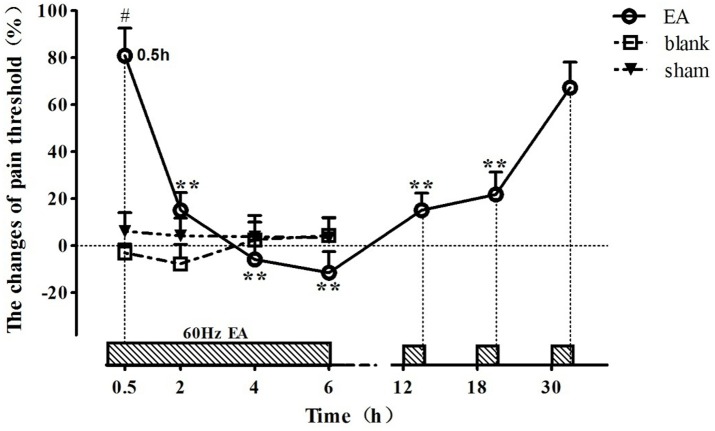
The time course of EA tolerance in goats (mean ± SD, %, *n* = 6). Goats were stimulated with 60 Hz EA. ^∗∗^Indicates the change of pain threshold is different (*p* < 0.01) from that at 0.5 h. ^#^Indicates there was difference (*p* < 0.01) in the change of pain threshold between the EA-treated goats and the blank or sham control-treated goats. Repeated ANOVA followed by Bonferroni’s post-test.

The change rates of pain threshold of the goats were shown in Figure 2 (relevant *df*, *F-*, and *p*-values of repeated ANOVA and Bonferroni’s post-test were shown in Supplementary Tables 1–3). The basal levels of the pain thresholds of blank control, sham control and EA-treated goats were 7.83 ± 2.43 V, 7.41 ± 2.13 V, and 7.86 ± 2.57 V, respectively. The change rates of pain threshold of the blank control and sham control did not change during the experiment. The change rate of pain threshold of EA-treated goats was 80.9 ± 11.7% at 0.5 h, showing potent analgesic effect of EA. And then it decreased (*p* < 0.01) at 2 h and fell to the lowest level (−11.4 ± 8.9%) at 6 h, showing the development of EAT. The change rate in pain threshold of the EA-treated goats increased at 12.5 h and returned to the higher level (67.3 ± 10.8%) at 30.5 h (which was not different from that at 0.5 h), indicating the recovery of EAT. The change rate of pain threshold of EA goats was higher than that of the blank or sham goats at 0.5 h (*p* < 0.01).

### The Effects of EA on Levels of ENK, CCK-8, and OFQ in the CNS of Goats

**FIGURE 3:**
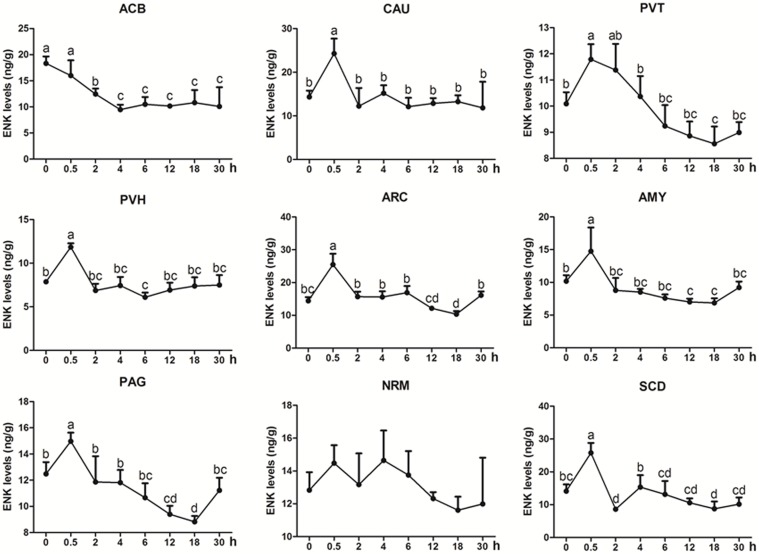
The effect of EA on ENK levels in the CNS of goats (mean ± SD, *n* = 6). ENK levels in the nucleus accumbens (ACB), the caudate nucleus (CAU), the paraventricular nucleus of the thalamus (PVT), the paraventricular nucleus of the hypothalamus (PVH), the arcuate nucleus (ARC), the amygdala (AMY), the periaqueductal gray (PAG), the nucleus raphe magnus (NRM), and the spinal cord dorsal horn (SCD). The values with different letters differ significantly (*p* < 0.05). One-way ANOVA followed by Bonferroni’s post-test.

ENK was measured in some analgesia-related nuclei or areas with its higher levels, including ACB, CAU, PVT, PVH, ARC, AMY, PAG, NRM, and SCD (Figure 3) (Relevant df, *F*-, and *p*-values of ANOVA were shown in Supplementary Table 4). The ENK level in ACB decreased (*p* < 0.01) at 2–4 h, and then remained lower level (*p* < 0.05) at 6–30 h. ENK in PAG, ARC, PVT, and AMY changed in a similar pattern; it increased to the peak level (*p* < 0.05) at 0.5 h, then gradually decreased and reached to the basal level (*p* > 0.05) at 2 h and to the lowest level (*p* < 0.05) at 12 or 18 h. ENK in CAU increased (*p* < 0.05) to the peak at 0.5 h, then decreased (*p* > 0.05) to the basal level at 2–30 h. ENK in NRM did not change (*p* > 0.05) during experiment. In SCD, ENK increased at 0.5 h (*p* < 0.05), and then decreased (*p* < 0.05) at 2 and 18 h. The change rates of pain thresholds were positively correlated with ENK levels in ACB (*r* = 0.477, *p* < 0.01), CAU (*r* = 0.672, *p* < 0.01), PVT (*r* = 0.674, *p* < 0.01), PVH (*r* = 0.809, *p* < 0.01), ARC (*r* = 0.815, *p* < 0.01), AMY (*r* = 0.761, *p* < 0.01), PAG (*r* = 0.722, *p* < 0.01), NRM (*r* = 0.325, *p* < 0.05), and SCD (*r* = 0.734, *p* < 0.01).

**FIGURE 4:**
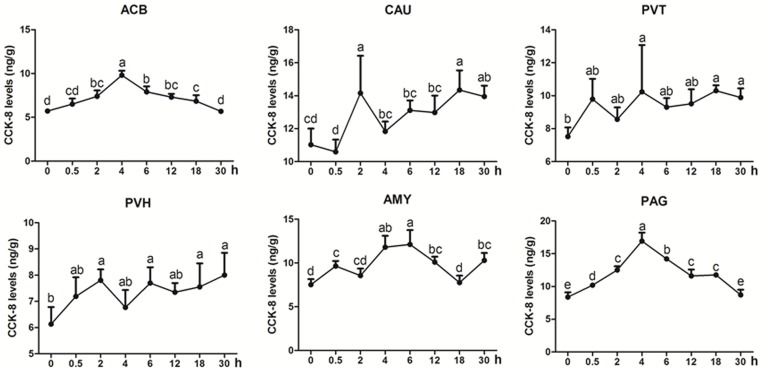
The effect of EA on CCK-8 levels in the CNS of goats (mean ± SD, *n* = 6). CCK-8 levels in the nucleus accumbens (ACB), the caudate nucleus (CAU), the paraventricular nucleus of the thalamus (PVT), the paraventricular nucleus of the hypothalamus (PVH), the amygdala (AMY), and the periaqueductal gray (PAG). The values with different letters differ significantly (*p* < 0.05). One-way ANOVA followed by Bonferroni’s post-test.

CCK-8 was measured in some analgesia-related nuclei or areas with its higher levels, including CAU, ACB, AMY, PVH, PVT, and PAG (Figure 4). CCK-8 levels increased (*p* < 0.05) with one or more peaks in the most measured nuclei or areas. EA increased (*p* < 0.05) CCK-8 in ACB (2–18 h) with the peak at 4 h and in PAG (0.5–18 h) with the peak at 4 h. CCK-8 fluctuated with its increment (*p* < 0.05) in CAU (2, 18, and 30 h), PVT (4, 18, and 30 h), PVH (2, 6, 18, and 30 h) and AMY (0.5, 4–12, and 30 h). The change rates of pain thresholds were negatively correlated with CCK-8 levels in CAU (*r* = −0.473, *p* < 0.01).

**FIGURE 5:**
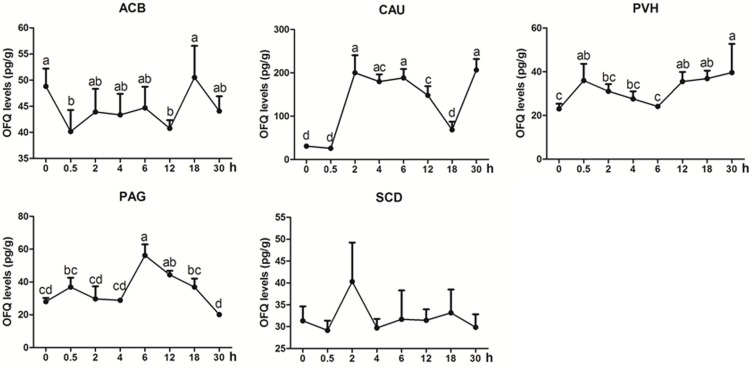
The effect of EA on OFQ levels in the CNS of goats (mean ± SD, *n* = 6). OFQ levels in the nucleus accumbens (ACB), the caudate nucleus (CAU), the paraventricular nucleus of the hypothalamus (PVH), the periaqueductal gray (PAG), and the spinal cord dorsal horn (SCD). The values with different letters differ significantly (*p* < 0.05). One-way ANOVA followed by Bonferroni’s post-test.

OFQ was measured in some analgesia-related nuclei or areas with its higher levels, including ACB, CAU, PVH, PAG, and SCD (Figure 5). OFQ levels in CAU increased with its peak (*p* < 0.01) at 2 h, and reduced to the pre-acupuncture level at 18 h, and then increased (*p* < 0.01) at 30 h again. EA induced increased OFQ (*p* < 0.05) in PVH (0.5 and 12–30 h) and PAG (6 and 12 h). In ACB, EA induced decreased OFQ levels (*p* < 0.05) at 0.5 and 12 h. There was no change (*p* > 0.05) in OFQ in SCD during the experiment. The change rates of pain thresholds were negatively correlated with OFQ levels in CAU (*r* = −0.335, *p* < 0.05) and ACB (*r* = −0.4776, *p* < 0.01).

**Table 3:** The effect of EA on levels of CCK gene expression in the CNS of goats (2^−ΔΔCt^, mean ± SD, n = 6).

Nuclei and areas	0 h	0.5 h	2 h	4 h	6 h	12 h	18 h	30 h
ACB	1.01 ± 0.09^a^	0.22 ± 0.17^bcd^	0.30 ± 0.06^bc^	0.16 ± 0.07^cd^	0.36 ± 0.08^b^	0.16 ± 0.04^cd^	0.13 ± 0.06^d^	0.19 ± 0.10^cd^
CAU	1.01 ± 0.12^bc^	1.37 ± 0.19^b^	0.70 ± 0.19^c^	1.23 ± 0.27^bc^	1.14 ± 0.29^bc^	2.03 ± 0.40^a^	0.98 ± 0.10^bc^	1.49 ± 0.48^ab^
PVT	0.96 ± 0.08^b^	0.14 ± 0.05^b^	0.11 ± 0.06^b^	0.13 ± 0.06^b^	0.19 ± 0.07^b^	2.59 ± 1.44^a^	0.15 ± 0.04^b^	0.32 ± 0.05^b^
PVH	1.01 ± 0.13^b^	0.82 ± 0.23^b^	0.97 ± 0.30^b^	1.27 ± 0.12^ab^	0.93 ± 0.26^b^	1.51 ± 0.33^a^	0.94 ± 0.22^b^	1.52 ± 0.36^a^
AMY	1.02 ± 0.13^ab^	1.26 ± 0.40^ab^	0.75 ± 0.25^ab^	1.39 ± 0.56^a^	0.77 ± 0.28^ab^	1.21 ± 0.44^ab^	0.83 ± 0.23^ab^	0.65 ± 0.20^b^
PAG	1.04 ± 0.11^c^	2.69 ± 0.23^a^	1.47 ± 0.24^bc^	1.87 ± 0.58^ab^	1.06 ± 0.13^bc^	0.89 ± 0.16^c^	2.55 ± 0.79^a^	2.43 ± 0.71^a^

### The Effects of EA on Gene Expression Levels of PENK, CCK, PNOC, MOR, CCKBR, and ORL1 in the CNS of Goats

During the induction of EAT, EA caused increased (*p* < 0.05) PENK mRNA levels in ARC (2 and 4 h), PAG (6 h) and SCD (6 h), but decreased (*p* < 0.05) PENK mRNA levels in ACB (4–6 h) and ARC (6 h) (Table 2) (Relevant df, *F*-, and *p*-values of ANOVA were shown in Supplementary Table 4). During the recovery of EAT, EA induced increased (*p* < 0.05) PENK mRNA levels in PVT, PVH, AYM, PAG, NRM, and SCD.

EA induced increased (*p* < 0.05) CCK mRNA levels in the most measured nuclei or areas, including CAU, PVT, PVH, AMY, and PAG at one or several time point(s) during the experiment (Table 4). Decreased (*p* < 0.05) CCK mRNA levels were found in ACB at 0.5–18 h.

**Table 4:** The effect of EA on the levels of PNOC gene expression in the CNS of goats (2^−ΔΔCt^, mean ± SD, n = 6).

Nuclei and areas	0 h	0.5 h	2 h	4 h	6 h	12 h	18 h	30 h
ACB	1.02 ± 0.07^c^	0.47 ± 0.09^d^	0.54 ± 0.06^d^	0.51 ± 0.10^d^	1.79 ± 0.27^a^	1.25 ± 0.50^bc^	1.15 ± 0.13^c^	1.63 ± 0.09^ab^
CAU	1.03 ± 0.12^bc^	0.73 ± 0.14^c^	0.68 ± 0.08^c^	0.95 ± 0.23^bc^	0.63 ± 0.16^c^	1.38 ± 0.37^a^	0.92 ± 0.17^bc^	1.10 ± 0.14^ab^
PVH	1.04 ± 0.12^bc^	0.96 ± 0.30^bc^	0.99 ± 0.24^bc^	0.92 ± 0.13^bc^	0.76 ± 0.24^c^	2.08 ± 0.51^a^	1.48 ± 0.40^ab^	1.20 ± 0.42^bc^
PAG	1.04 ± 0.11	0.72 ± 0.08	0.91 ± 0.20	1.09 ± 0.22	0.90 ± 0.24	0.93 ± 0.25	1.06 ± 0.36	1.17 ± 0.43
SCD	0.99 ± 0.12^bc^	0.62 ± 0.34^c^	0.64 ± 0.29^c^	2.09 ± 0.39^b^	1.11 ± 0.73^bc^	3.94 ± 1.45^a^	1.44 ± 0.57^bc^	0.59 ± 0.21^c^

EA induced increased (*p* < 0.05) PNOC mRNAs in the most measured nuclei or areas including CAU, PVH and SCD at one or several time point(s) during the experiment (Table 5). PNOC mRNA levels fluctuated in ACB during the experiment. No difference (*p* > 0.05) in PNOC mRNA levels was found in PAG.

**Table 5:** The effect of EA on levels of CCKBR gene expression in the CNS of goats (2^−ΔΔCt^, mean ± SD, n = 6).

Nuclei and areas	0 h	0.5 h	2 h	4 h	6 h	12 h	18 h	30 h
ACB	1.02 ± 0.16^c^	1.42 ± 0.22^bc^	1.83 ± 0.44^bc^	1.63 ± 0.43^bc^	3.63 ± 2.83^ab^	1.79 ± 0.53^bc^	4.92 ± 1.12^a^	4.28 ± 0.94^a^
CAU	1.05 ± 0.12^b^	1.72 ± 1.04^ab^	1.42 ± 0.70^ab^	2.58 ± 1.16^ab^	3.16 ± 2.08^a^	1.83 ± 1.07^ab^	2.04 ± 0.64^ab^	2.69 ± 0.99^ab^
PVT	1.03 ± 0.11^b^	0.34 ± 0.23^b^	0.14 ± 0.06^b^	0.36 ± 0.21^b^	0.32 ± 0.13^b^	0.84 ± 0.66^b^	18.62 ± 14.89^a^	1.46 ± 0.26^b^
PVH	1.03 ± 0.08^b^	1.87 ± 1.27^b^	0.87 ± 0.48^b^	1.70 ± 0.76^b^	0.74 ± 0.36^b^	7.89 ± 3.04^a^	2.10 ± 1.02^b^	2.46 ± 2.01^b^
AMY	1.05 ± 0.09	0.67 ± 0.36	1.03 ± 0.39	1.43 ± 0.78	0.61 ± 0.17	1.37 ± 0.62	0.92 ± 0.64	1.23 ± 0.67
PAG	0.98 ± 0.10^a^	0.29 ± 0.12^bc^	0.28 ± 0.08^bc^	1.03 ± 0.68^a^	0.12 ± 0.04^c^	0.81 ± 0.55^ab^	0.48 ± 0.33^bc^	0.44 ± 0.21^bc^

EA induced fluctuated CCKBR mRNA levels (Table 5). Increased (*p* < 0.05) OPRL1 mRNA levels were found in PVH (12 h), ACB (18 h), and CAU (30 h) (Table 6). OPRL1 mRNAs decreased (*p* < 0.05) in SCD at 0.5, 2, 6, 18, and 30 h. It fluctuated in PAG. Decreased (*p* < 0.05) MOR mRNA levels were observed in PAG at 4 h (Tables 2–7). MOR mRNAs increased (*p* < 0.05) in PVH (12 h), NRM (12 h), PVT (18 h), ARC (18 h), ACB (18 and 30 h), and CAU (30 h). MOR mRNAs in SCD were found to decrease (*p* < 0.05) at 0.5, 2, 6, 18, and 30 h and to increase (*p* < 0.05) at 12 h.

**Table 6:** The effect of EA on levels of OPRL1 gene expression in the CNS of goats (2^−ΔΔCt^, mean ± SD, n = 6).

Nuclei and areas	0 h	0.5 h	2 h	4 h	6 h	12 h	18 h	30 h
ACB	1.04 ± 0.12^b^	0.75 ± 0.19^b^	1.32 ± 0.27^ab^	1.25 ± 0.33^ab^	0.79 ± 0.20^b^	1.29 ± 0.34^ab^	1.84 ± 0.44^a^	1.33 ± 0.44^ab^
CAU	1.01 ± 0.14^b^	1.86 ± 1.14^b^	1.20 ± 0.71^b^	3.01 ± 2.16^ab^	2.63 ± 2.38^ab^	4.00 ± 2.06^ab^	1.99 ± 0.88^b^	6.41 ± 4.02^a^
PVH	1.04 ± 0.06^b^	1.78 ± 0.94^b^	0.71 ± 0.49^b^	1.45 ± 0.65^b^	0.70 ± 0.15^b^	5.03 ± 2.06^a^	2.32 ± 1.37^b^	1.66 ± 1.01^b^
PAG	1.04 ± 0.11^cd^	0.63 ± 0.23^de^	0.54 ± 0.15^e^	1.43 ± 0.25^ab^	0.48 ± 0.14^e^	1.86 ± 0.36^a^	0.80 ± 0.29^cd^	1.06 ± 0.18^bc^
SCD	1.00 ± 0.13^a^	0.09 ± 0.07^b^	0.11 ± 0.08^b^	1.08 ± 0.63^a^	0.20 ± 0.10^b^	0.59 ± 0.28^ab^	0.26 ± 0.09^b^	0.16 ± 0.13^b^

**Table 7:** The effect of EA on levels of MOR gene expression in the CNS of goats (2^−ΔΔCt^, mean ± SD, n = 6).

Nuclei and areas	0 h	0.5 h	2 h	4 h	6 h	12 h	18 h	30 h
ACB	1.01 ± 0.11^c^	1.15 ± 0.18^bc^	1.18 ± 0.09^bc^	1.04 ± 0.78^c^	1.21 ± 0.37^bc^	1.07 ± 0.21^c^	2.14 ± 0.55^a^	1.88 ± 0.33^ab^
CAU	1.03 ± 0.13^bc^	1.02 ± 0.22^c^	0.65 ± 0.18^c^	0.75 ± 0.28^c^	0.94 ± 0.27^bc^	0.96 ± 0.23^bc^	1.37 ± 0.37^ab^	1.50 ± 0.15^a^
PVT	1.01 ± 0.12^b^	1.11 ± 0.29^b^	0.59 ± 0.11^b^	0.74 ± 0.09^b^	0.65 ± 0.40^b^	0.71 ± 0.29^b^	6.26 ± 1.46^a^	0.90 ± 0.27^b^
PVH	1.01 ± 0.12^cd^	1.07 ± 0.09^cd^	0.74 ± 0.24^cd^	0.59 ± 0.16^d^	0.65 ± 0.10^d^	1.63 ± 0.31^a^	1.20 ± 0.44^bc^	1.35 ± 0.36^ab^
ARC	1.03 ± 0.11^b^	1.02 ± 0.25^b^	0.39 ± 0.14^b^	0.42 ± 0.09^b^	1.70 ± 0.40^b^	0.94 ± 0.15^b^	9.80 ± 2.47^a^	0.45 ± 0.17^b^
AMY	1.04 ± 0.13^a^	1.19 ± 0.28^a^	0.58 ± 0.25^a^	0.56 ± 0.20^a^	0.60 ± 0.27^a^	1.05 ± 0.29^a^	0.79 ± 0.28^a^	0.89 ± 0.27^a^
PAG	1.01 ± 0.10^ab^	1.14 ± 0.35^a^	0.52 ± 0.39^bc^	0.37 ± 0.22^c^	0.50 ± 0.33^bc^	0.74 ± 0.25^ab^	0.77 ± 0.25^ab^	0.75 ± 0.16^ab^
NRM	1.02 ± 0.07^b^	1.04 ± 0.08^b^	0.18 ± 0.04^b^	0.54 ± 0.15^b^	0.21 ± 0.12^b^	3.34 ± 1.54^a^	0.25 ± 0.10^b^	0.29 ± 0.12^b^
SCD	1.05 ± 0.15^b^	0.09 ± 0.02^c^	0.10 ± 0.02^c^	0.99 ± 0.40^b^	0.23 ± 0.03^c^	2.47 ± 0.47^a^	0.43 ± 0.09^c^	0.35 ± 0.41^c^

## Discussion

EA has been extensively used for treating various diseases, especially pain disorders. However, it also can provoke EAT, which is a negative effect for its application. Acute EAT model has been set up for studying EAT mechanism. Li et al. (1980) stimulated rats alternatively using 2 and 15 Hz EA for 6 sections (each section consists of 30 min EA and 30 min non-EA interval), and found that the change in pain threshold was 89 ± 12% at 1 h, followed by 61 ± 11% at 3 h and 21 ± 9% at 6 h, showing that intermittent EA can provoke acute EAT. Xie et al. (1981) continually stimulated rats alternatively using 2 and 15 Hz EA for 6 h, and found that the change in pain threshold was 133 ± 8% at 10 min, followed by 53 ± 18% at 2 h, 4 ± 11% at 4 h, and –11 ± 7% at 6 h. Tian et al. (1998) used 100 Hz EA to stimulate rats for consecutive 6 h, and found that the pain threshold decreased at 1 h and approximated to the level before EA at 6 h. In the present study, 60 Hz EA was used to stimulate goats for consecutive 6 h. The change in pain threshold was reduced from 89.9 ± 11.7% at 0.5 h to –11.4 ± 8.9% at 6 h, indicating a rapid formation of EAT. The result in our experiment is similar to the report by Xie et al. (1981). The recovery time of EAT may vary with different EAT modalities. Li et al. (1980) reported the time for pain threshold recovery induced by intermittent EA in rats was 24 h. The study of Xie et al. (1981) showed that continual EA-induced pain threshold in rats took 32 h to return to the pre-EA level. In our study, the recovery time of EAT induced by continual EA (60 Hz) was 24 h, which was shorter than the reports by Xie et al. (1981). Such discrepancy is probably due to frequency or species variation.

Numerous studies confirmed that EOPs in the CNS participate in EA-induced analgesia regulation (Sun and Han, 1989; Staff, 2014; Cui and Ding, 2016; Hu et al., 2018). However, EOP expression patterns in some nuclei or areas in the development and recovery of EAT have been rarely reported. Because ENK is extensively distributed in the analgesia-related nuclei or areas and plays an important role in the regulation of EA-induced analgesia, it can be selected as a representative of EOPs for this study. In the present trial, pain thresholds were consistent with those of ENK levels in the most measured nuclei or areas of goats; both increased at 0.5 h, then rapidly decreased at 2 h, fell to the lowest level, and returned to the pre-EA level at 30 h. Statistics analysis showed that the changes in pain threshold were positively correlated with ENK levels in ACB, CAU, PVT, PVH, ARC, AMY, PAG, NRM, and SCD; ENK levels in these nuclei or areas had reversely change with EAT extent. It is believed that increased ENK mRNAs are contributory to replenishing the consumed ENK. Zhu et al. (1993) observed the increased PENK mRNAs in ACB, CAU, and PAG at 10 h after EA were terminated in rats. Cheng et al. (2013) showed that PENK mRNA levels increased in ACB, CAU, AMY, PVH, and PAG in the CNS of goats at 4 h after EA termination. In this study, PENK mRNA levels decreased or did not change in ACB, CAU, AMY, PVT, and PAG at 4 h, indicating continuous EA has an inhibitory effect on PENK mRNA expression in some nuclei or areas.

It has been found that some neuromodulators, such as CCK-8 and OFQ, have a potent anti-opioid effect. Therefore, the roles of these anti-opioid peptides in EAT have attracted more attention. Han et al. (1985) injected CCK-8 into the lateral cerebral ventricle or spinal subarachnoid space in rats, and found that CCK-8 dose-dependently antagonized EA-induced analgesic effect (15 Hz, 10 min). They further confirmed that intracerebroventricular (ICV) or intrathecal injection of antiserum against CCK-8 post-poned or reversed the EAT in rats (Han et al., 1986). Bian et al. (1993) also confirmed that ICV injection of CCK-8 antiserum effectively reversed EAT. In the present study, CCK-8 levels in CAU were negatively correlated with EA analgesic effects. These studies indicate that endogenous CCK-8 may participate in the formation of EAT. Sun et al. (1995) stimulated rats with 15 Hz EA for consecutive 6 h, and found that CCK-8-immunoreaction increased in the spinal perfusate and PAG at 2 h, and its mRNA increased in the brain at 8 h. In our study, continuous EA caused increased CCK-8 levels in ACB, CAU, PVT, PVH, AMY, and PAG with the peak at 2–4 h, and increased CCK-8 mRNA levels in CAU, PVT, PVH, and PAG at 4–12 h, which is similar to the report by Sun et al. (1995). It is found from the above studies that EA-induced release of CCK-8 lagged the release of ENK in the CNS. Shen et al. (1995) reported that naloxone, an antagonist of EOP, inhibited EA-induced increase in CCK-8 in the CSF. Therefore, EA-induced increase in CCK-8 in our experiment may be regarded as a negative feedback on the effects of opioid-like substances.

OFQ, an endogenous ligand of ORL1, has been demonstrated to block antinociceptive effects induced by opioids (Kamei et al., 1999; Chen et al., 2007). It has been reported that both 2 Hz and 100 Hz EA stimulations enhanced analgesic effects in OFQ knock-out mice than in wild-type control mice (Wan et al., 2009). Microinjection of OFQ into rat’s PAG remarkably antagonizes EA analgesia in a dose-related manner (Zhao, 2008). These results indicate that OFQ has an antagonist effect on EA analgesia. However, the expression pattern of OFQ in the development and recovery of EAT was less well understood. Tian et al. (1998) found that ICV injection of the antibody to OFQ reversed the decreased pain threshold induced by 6 h of EA. In our experiment, continuous EA induced increased OFQ levels in PVH at 0.5 h and in CAU and PAG at 2–6 h, and increased OFQ mRNA levels in the most measured nuclei or areas at 12 h. These results suggest that EA accelerates the release and biosynthesis of OFQ (like CCK-8) in the CNS to antagonize the effect of opioids, which may serve as a delayed negative feedback on opioid analgesia.

It was discovered that prolonged or repeated EA resulted in the development of tolerance to EA and the cross-tolerance to morphine. This shows that the exhaustion of ENK during EA cannot completely explain EAT. Since opioid peptides exert biological functions through their receptors, opioid receptors may have a critical role in the development of EAT. MOR is one of classic opioid receptors with a high affinity for ENK and beta-endorphin (Han J. et al., 1981; Chen and Han, 1992a; Huang et al., 2000). Ni et al. (1987) reported that opioid receptors decreased by more than 30% in nuclei of EAT rats’ brain, including NRM, PAG, AMY, ACB, PVH, PVT, and CAU. Cheng et al. (2013) observed that 30 min of EA induced increased MOR mRNA levels at 4 h in CAU, PVH, ARC, AMY, PAG, and NRM. However, in the present study, 6 h of EA elicited decreased or unchanged MOR mRNA levels at 2–6 h in the most measured nuclei or areas. These results indicate that continuous EA inhibits MOR expression, which may contribute to the development of EAT. Little is known about the role of CCK receptors and OPLR1 in the development and recovery of EAT. Our results showed the mRNA expression of CCKBR and OPLR1 fluctuated during EA and increased at 12 h in the most measured nuclei or areas. Studies have demonstrated that CCK-8 can combine CCK receptors (especially CCKBR) in the specific sites of the CNS to interfere with the functions of MOR and Kappa opioid receptors, resulting in anti-analgesic effects (Wang et al., 1990; Shen et al., 1995; Huang et al., 2007). Mogil et al. (1996) demonstrated that OFQ was able to antagonize opioid analgesia mediated by opioid receptors in the brain. CCKBR and OPLR1 may exert their influence on opioid receptors through several ways. Studies have demonstrated that CCK and OFQ can induce increased intracellular IP3, antagonize the suppression of Calcium channels mediated by MOR, and reduce the biding affinity of ligands to MOR (Han, 2001; Wang et al., 2006). However, the molecular mechanisms by which these receptors regulate EAT need to be further studied.

EA induces the release of the neuromodulators in the specific nuclei or regions, thereby exerts the analgesic modification. The opioid peptide (ENK) or anti-opioid peptides (CCK-8 and OFQ) and their receptors (MOR, CCKBR, and OPRL1) were distributed in different nuclei or regions of the CNS. Therefore, each of these substances was detected in specific nuclei or areas with its higher levels in this trial. Our results showed that EA-induced expressing levels of opioid peptides, anti-opioid peptides, and their receptors were fluctuated in some nuclei, indicating the complex regulations of these neuromodulators and nuclei in the development and recovery of EAT. Although the present study displayed the dynamic expression patterns of opioid and anti-opioid peptides in a wide range of the brain regions, the interplay between them during the development and recovery of EAT needs to be investigated. Anyhow, our results provide an important foundation for selecting proper time points to interfere with or for understanding the changes of these neuromodulators in the nuclei or areas in the development of EAT.

## Conclusion

EAT was induced by 60 Hz EA for consecutive 6 h in goats. Continuous EA induced increased ENK levels at 0.5 h and decreased ENK levels at 2–18 h. EA provoked increased CCK-8 and OFQ levels in most measured nuclei or areas. The change rates of pain thresholds were correlated positively with ENK levels in all the measured nuclei or areas, but negatively with CCK-8 levels in CAU with OFQ levels in ACB and CAU. Continuous EA inhibited mRNA expression of PENK and MOR, but increased mRNA expression of CCK and OFQ and their receptors in some analgesia-related nuclei or areas. These results suggest that the development and recovery of EAT may be associated with the specific expression patterns of ENK, CCK-8, and OFQ and their receptors in the analgesia-related nuclei or areas.

## Author Contributions

MD contributed to conception and design of the study. JW and ZQ performed animal experiments, collected samples and accomplished the laboratory investigations. YD, JW, and ZQ performed acquisition of data. YD, JW, and SN conducted data analysis and interpretation of data. JW and ZQ drafted the manuscript. MD, YD, ZQ, and JW revised the manuscript. All authors read and approved the final manuscript.

## Conflict of Interest Statement

The authors declare that the research was conducted in the absence of any commercial or financial relationships that could be construed as a potential conflict of interest.

## Abbreviations

ACB: the nucleus accumbens
AMY: the nucleus amygdala
ARC: the arcuate nucleus
CAU: the caudate nucleus
CCK-8: cholecystokinin octapeptide
CCKBR: CCK B type receptor
CNS: the central nervous system
EA: electroacupuncture
EAT: electroacupuncture tolerance
ENK: enkephalin
EOPs: endogenous opioid peptides
ICV: intracerebroventricular
MOR: μ opioid receptor
NRM: the nucleus raphe magnus
OFQ: orphanin FQ
OPRL1: opioid receptor-like 1 receptor
PAG: the periaqueductal gray
PENK: preproenkephalin
PNOC: prepronociceptin
PVH: the paraventricular nucleus of the hypothalamus
PVT: the paraventricular nucleus of the thalamus
SCD: spinal cord dorsal horn
