# Supplementary material for: The Expressing Patterns of Opioid Peptides, Anti-opioid Peptides and Their Receptors in the Central Nervous System Are Involved in Electroacupuncture Tolerance in Goats
Source: Front Neurosci. 2018 Dec 13;12:902. doi: 10.3389/fnins.2018.00902 (PMC6300483; doi:10.3389/fnins.2018.00902)
Supplement: Supplementary file 1 [file Table_1.docx]

| Supplementary Table 1: Repeated ANOVA for change rates of pain thresholds in goats | | | |
| --- | --- | --- | --- |
| Comparison | df | F | p |
| Among 0.5, 2, 4, 6, 12.5, 18.5 and 30.5 h in EA group | 6 | 90.463 | 0.000 |
| Among Blank, Sham and EA groups at 0.5, 2, 4 and 6 h |  |  |  |
| Main effect of time | 3 | 51.835 | 0.000 |
| Main effect of group | 2 | 38.820 | 0.000 |
| Cross effect of time * group | 6 | 57.733 | 0.000 |

F, p and df values for the comparison of change rates of pain thresholds in goats among Blank, Sham and EA groups at 0.5, 2, 4 and 6 h, and among 0.5, 2, 4, 6, 12.5, 18.5 and 30.5 h in EA group were analyzed with repeated ANOVA.
