# Supplementary material for: The Expressing Patterns of Opioid Peptides, Anti-opioid Peptides and Their Receptors in the Central Nervous System Are Involved in Electroacupuncture Tolerance in Goats
Source: Front Neurosci. 2018 Dec 13;12:902. doi: 10.3389/fnins.2018.00902 (PMC6300483; doi:10.3389/fnins.2018.00902)
Supplement: Supplementary file 2 [file Table_2.docx]

| \| Supplementary Table 2: P values for the comparison of pain thresholds of change rates between EA and Blank or Sham groups in goats \| \| \| \| \| --- \| --- \| --- \| --- \| \| Time point (h) \| Groups \| \| P value \| \| \| 0.5 \| EA \| Blank \| 0.000 \| \| Sham \| 0.000 \| \| 2 \| EA \| Blank \| 0.000 \| \| Sham \| 0.084 \| \| 4 \| EA \| Blank \| 0.327 \| \| Sham \| 0.198 \| \| 6 \| EA \| Blank \| 0.013 \| \| Sham \| 0.026 \| |  |  |  |
| --- | --- | --- | --- | --- | --- | --- | --- | --- | --- | --- | --- | --- | --- | --- | --- | --- | --- | --- | --- | --- | --- | --- | --- | --- | --- | --- | --- | --- | --- | --- | --- | --- | --- | --- | --- |

P values for the comparison of change rates of pain thresholds in goats between EA and Blank or Sham groups at specific time points (0.5, 2, 4 and 6 h) were analyzed with Bonferroni’s post-test.
