# Supplementary material for: The Expressing Patterns of Opioid Peptides, Anti-opioid Peptides and Their Receptors in the Central Nervous System Are Involved in Electroacupuncture Tolerance in Goats
Source: Front Neurosci. 2018 Dec 13;12:902. doi: 10.3389/fnins.2018.00902 (PMC6300483; doi:10.3389/fnins.2018.00902)
Supplement: Supplementary file 3 [file Table_3.docx]

| Supplementary Table 3: P values for the comparison of change rates of pain thresholds between different time points in EA-treated goats | | |
| --- | --- | --- |
| Time points (h) | | p value |
|  |  |  |
| 0.5 | 2 | 0.000 |
|  | 4 | 0.000 |
|  | 6 | 0.000 |
|  | 12.5 | 0.000 |
|  | 18.5 | 0.000 |
|  | 30.5 | 0.266 |

P values for the comparison of change rates of pain threshold between 0.5 and 2, 4, 6, 12.5, 18.5 or 30.5 h in EA-treated goats were analyzed with Bonferroni’s post-test.
