# Supplementary material for: The Expressing Patterns of Opioid Peptides, Anti-opioid Peptides and Their Receptors in the Central Nervous System Are Involved in Electroacupuncture Tolerance in Goats
Source: Front Neurosci. 2018 Dec 13;12:902. doi: 10.3389/fnins.2018.00902 (PMC6300483; doi:10.3389/fnins.2018.00902)
Supplement: Supplementary file 4 [file Table_4.docx]

| Supplementary Table 4: ANOVA for protein or mRNA levels of opioid peptides, anti-opioid peptides or their receptors at different time points in the CNS of EA-treated goats | | | | | | | | | | | | | | | | | | | |
| --- | --- | --- | --- | --- | --- | --- | --- | --- | --- | --- | --- | --- | --- | --- | --- | --- | --- | --- | --- |
| Nuclei and areas | df | ENK | | CCK-8 | | OFQ | | PENK mRNA | | MOR mRNA | | CCK mRNA | | CCKBR mRNA | | PNOC mRNA | | OPRL1 mRNA | |
|  |  | F | p | F | p | F | p | F | p | F | p | F | p | F | p | F | p | F | p |
| CAU | 7 | 10.463 | 0.000 | 9.769 | 0.000 | 74.151 | 0.000 | 3.046 | 0.012 | 8.576 | 0.000 | 11.959 | 0.000 | 2.438 | 0.035 | 9.731 | 0.000 | 4.599 | 0.001 |
| ACB | 7 | 14.842 | 0.000 | 37.139 | 0.000 | 4.787 | 0.001 | 8.733 | 0.000 | 6.976 | 0.000 | 59.930 | 0.000 | 9.669 | 0.000 | 33.259 | 0.000 | 6.469 | 0.000 |
| PVH | 7 | 28.228 | 0.000 | 5.017 | 0.000 | 6.392 | 0.000 | 17.462 | 0.000 | 11.309 | 0.000 | 6.832 | 0.000 | 15.431 | 0.000 | 10.502 | 0.000 | 10.760 | 0.000 |
| PAG | 7 | 19.790 | 0.000 | 91.296 | 0.000 | 19.673 | 0.000 | 16.480 | 0.000 | 5.577 | 0.000 | 15.906 | 0.000 | 6.049 | 0.000 | 1.772 | 0.120 | 25.215 | 0.000 |
| PVT | 7 | 18.971 | 0.000 | 3.537 | 0.005 |  |  | 117.547 | 0.000 | 69.979 | 0.000 | 17.106 | 0.000 | 8.769 | 0.000 |  |  |  |  |
| AMY | 7 | 15.596 | 0.000 | 18.719 | 0.000 |  |  | 6.161 | 0.000 | 5.677 | 0.000 | 3.972 | 0.002 | 1.995 | 0.080 |  |  |  |  |
| SCD | 7 | 27.848 | 0.000 |  |  | 3.277 | 0.008 | 4.190 | 0.002 | 52.663 | 0.000 |  |  |  |  | 18.323 | 0.000 | 9.198 | 0.000 |
| ARC | 7 | 39.429 | 0.000 |  |  |  |  | 87.288 | 0.000 | 76.452 | 0.000 |  |  |  |  |  |  |  |  |
| NRM | 7 | 3.002 | 0.012 |  |  |  |  | 8.167 | 0.000 | 22.226 | 0.000 |  |  |  |  |  |  |  |  |
|  |  |  |  |  |  |  |  |  |  |  |  |  |  |  |  |  |  |  |  |

F, p and df values for protein or mRNA levels of opioid peptides, anti-opioid peptides or their receptors among 0, 0.5, 2, 4, 6, 12, 18 and 30 h in the CNS of EA-treated goats were analyzed with one-way ANOVA.

ENK: enkephalin; CCK-8: cholecystokinin octapeptide; OFQ: orphanin FQ, PENK: preproenkephalin; MOR:µ- opioid receptor; CCK: cholecystokinin; CCKBR: CCKB receptor; PNOC: prepronociceptin; OPRL1: opioid receptor-like1 receptor; ACB: the nucleus accumbens; CAU: the caudate nucleus; PVT: the paraventricular nucleus of the thalamus; PVH: the paraventricular nucleus of the hypothalamus, ARC: the arcuate nucleus, AMY: the amygdala; PAG: the periaqueductal grey; NRM: the nucleus raphe magnus; SCD: the spinal cord dorsal horn.
